# Viral Glycoproteins Induce NLRP3 Inflammasome Activation and Pyroptosis in Macrophages

**DOI:** 10.3390/v13102076

**Published:** 2021-10-15

**Authors:** Hannah S. Eisfeld, Alexander Simonis, Sandra Winter, Jason Chhen, Luisa J. Ströh, Thomas Krey, Manuel Koch, Sebastian J. Theobald, Jan Rybniker

**Affiliations:** 1 Department I of Internal Medicine, Faculty of Medicine and University Hospital Cologne, University of Cologne, 50931 Cologne, Germany; hannah.eisfeld@uk-koeln.de (H.S.E.); alexander.simonis@uk-koeln.de (A.S.); sandra.winter@uk-koeln.de (S.W.); jason.chhen@uk-koeln.de (J.C.); 2 Center for Molecular Medicine Cologne (CMMC), Faculty of Medicine and University Hospital Cologne, University of Cologne, Robert-Koch-Strasse 21, 50931 Cologne, Germany; manuel.koch@uni-koeln.de; 3 Institute of Virology, Hannover Medical School, 30625 Hannover, Germany; Stroeh.Luisa@mh-hannover.de (L.J.S.); krey@biochem.uni-luebeck.de (T.K.); 4 Center of Structural and Cell Biology in Medicine, Institute of Biochemistry, University of Luebeck, 23562 Luebeck, Germany; 5 German Center for Infection Research (DZIF), Partner Site Hamburg-Lübeck-Borstel-Riems, 22607 Hamburg, Germany; 6 Excellence Cluster 2155 RESIST, Hannover Medical School, 30625 Hannover, Germany; 7 Centre for Structural Systems Biology (CSSB), 22607 Hamburg, Germany; 8 Institute for Dental Research and Oral Musculoskeletal Biology and Center for Biochemistry, Faculty of Medicine and University Hospital Cologne, University of Cologne, Joseph-Stelzmann-Strasse 52, 50931 Cologne, Germany; 9 German Center for Infection Research (DZIF), Partner Site Bonn-Cologne, 50931 Cologne, Germany

**Keywords:** NLRP3, IL-1β, pyroptosis, viral glycoproteins, innate immunity, macrophages

## Abstract

Infections with viral pathogens are widespread and can cause a variety of different diseases. In-depth knowledge about viral triggers initiating an immune response is necessary to decipher viral pathogenesis. Inflammasomes, as part of the innate immune system, can be activated by viral pathogens. However, viral structural components responsible for inflammasome activation remain largely unknown. Here we analyzed glycoproteins derived from SARS-CoV-1/2, HCMV and HCV, required for viral entry and fusion, as potential triggers of NLRP3 inflammasome activation and pyroptosis in THP-1 macrophages. All tested glycoproteins were able to potently induce NLRP3 inflammasome activation, indicated by ASC-SPECK formation and secretion of cleaved IL-1β. Lytic cell death via gasdermin D (GSDMD), pore formation, and pyroptosis are required for IL-1β release. As a hallmark of pyroptosis, we were able to detect cleavage of GSDMD and, correspondingly, cell death in THP-1 macrophages. CRISPR-Cas9 knockout of NLRP3 and GSDMD in THP-1 macrophages confirmed and strongly support the evidence that viral glycoproteins can act as innate immunity triggers. With our study, we decipher key mechanisms of viral pathogenesis by showing that viral glycoproteins potently induce innate immune responses. These insights could be beneficial in vaccine development and provide new impulses for the investigation of vaccine-induced innate immunity.

## 1. Introduction

Viral infections and infection-associated complications represent a major global health problem. The diversity of viruses and viral biology is reflected by the broad range of clinical symptoms ranging from asymptomatic, to chronic to acute and fulminant courses. In addition to the spectrum of severity, viral diseases differentiate highly in contagiosity, including a low infectivity to highly pathogenic viruses, which can cause global pandemics affecting millions of people. In addition to the ongoing HIV pandemic, several outbreaks have aroused public attention in the last years as the SARS epidemic in 2003 or several Ebola outbreaks in West Africa between 2014 to 2016. Furthermore, the world is facing an ongoing coronavirus disease 19 (COVID-19) pandemic with over 220 million confirmed cases and over 4 million deaths worldwide [1].

COVID-19 is caused by the severe acute respiratory syndrome coronavirus 2 (SARS-CoV-2). SARS-CoV-2 is a single-stranded positive-sense RNA virus and belongs to the ß-coronavirus subfamily [2]. The most common symptoms in patients are fever, cough, and fatigue [3]. As some patients fail to control viral infection, SARS-CoV-2 can cause severe viral pneumonia leading to respiratory failure [4]. The genome of SARS-CoV-2 shows nearly 80% sequence identity to SARS-CoV, which caused an epidemic in 2003 affecting approximately 8000 people with 774 deaths [2,5]. Similar to COVID-19, patients presented fever, cough, myalgia, and dyspnea and developed in some cases atypical pneumonia [5].

Hepatitis C virus (HCV) and human Cytomegalovirus (HCMV) are examples of viruses causing mostly chronic infections. HCV is an enveloped virus with a positive-sense single-stranded RNA genome [6]. The virus is transmitted through exchange of body fluids. In 20% of infected individuals, HCV causes an acute infection with subsequent viral clearance. However, in 80% of patients the infection progresses to a chronic state and patients are at risk for liver cirrhosis and other complications [6]. HCMV is an enveloped virus with a double-stranded DNA genome. HCMV infections are mainly asymptomatic but can also cause severe infections in immune compromised hosts or congenital infections [7,8].

In the defense against viral pathogens, the immune system of the host has developed several strategies to avert viral entry and viral reproduction. In the last years, the adaptive immune system, including the cellular response of lymphocyte to viral infections has been the focus in the research of host–pathogen interactions and viral pathogenesis. However, the innate immunity plays an underestimated role in viral defense [9]. Components of the innate immune system represent the first line of defense and are essential for the establishment of full antiviral immunity. Here, inflammasomes play a crucial role for host immune responses. They are intracellular multi-protein complexes essential for the secretion of the inflammatory cytokines Interleukin-1ß (IL-1ß) and IL-18, required for the recruitment of immune cells [10]. The inflammasome activation process is initiated through the recognition of pathogen-associated molecular patterns (PAMPs) by pattern recognition receptors (PRRs). PAMPs are molecular motifs conserved within a class of microbes, e.g., lipopolysaccharides (LPS) [10]. This first step leads to the activation of the nuclear factor kappa-light-chain-enhancer of activated B cells (NFkB), a transcription factor which upregulates the expression of pro-IL-1ß and NLRP3 [10]. In a second step, an activation signal is needed to induce complete inflammasome activation. Activation signals are PAMPs or danger-associated molecular patterns (DAMPs), such as aberrant ionic fluxes through viroporins [10]. The most investigated inflammasome is the NOD-, LRR-, and pyrin domain-containing protein 3 (NLRP3) inflammasome, a multi-protein complex, which upon activation triggers the oligomerization of the adaptor protein ASC. After inflammasome assembly, pro-caspase-1 is auto-cleaved and subsequently processes pro-Interleukin-1ß (pro-IL-1ß) proteolytically [10]. Further, the NLRP3 inflammasome induces pyroptosis, a form of pro-inflammatory and regulated necrotic cell death. Here, gasdermin D (GSDMD) is cleaved by caspase-1 and forms pores in the cellular membrane leading to cell lysis [11]. Inflammasomes are highly important for a successful antiviral response; however, they can also contribute to the immunopathology and hyperinflammatory states [9,12,13]. Therefore, a better understanding of the underlying mechanisms leading to inflammasome activation in viral diseases is of particular importance. In fact, there remain several open questions on how viral pathogens induce an inflammasome response in cells of the innate immune system and which virus derived structural components function as triggers.

Activation of immune cells by viral pathogens is mainly mediated by surface proteins including glycoproteins, which interfere with the host cell. Glycoproteins play a crucial role in cellular entry and are known as important antigens for host immune responses [14,15,16]. In SARS-CoV and SARS-CoV-2 the spike protein mediates cellular entry by binding to the angiotensin-converting enzyme 2 (ACE2) [14]. The spike protein of SARS-CoV-2 (SP-CoV-2) has generated particular interest as the main target in vaccine development [17]. In hepatitis C infections viral entry is mediated through the interactions of the surface proteins E1 and E2 (E2 HCV) with host cell receptors. These glycoproteins are crucial factors of HCV pathogenicity [16]. In HCMV infections, several structures contributing to cellular entry are known. One important antigen is the glycoprotein B (gB HCMV), which is responsible for membrane fusion during viral entry [15]. Commonly, all proteins represent potent viral antigens, which are targeted by the cellular and humoral immune system and represent important vaccine antigens [18,19,20,21]. However, mechanisms of activation of cells associated with innate immunity by these glycoproteins is poorly understood. Recently, we were able to show that SP-CoV-2 potently triggers inflammasome formation and consequently IL-1ß secretion in macrophages derived from COVID-19 patients, but not healthy individuals. Epigenetic changes and gene expression profiles from COVID-19 macrophages suggest an innate memory response of in vivo primed and SARS-CoV-2 experienced macrophages [22]. Still, the question remains if other viral glycoproteins can have similar effects.

Within this manuscript, we analyze the role of several viral glycoproteins as innate immunity triggers in vitro. All studied viral glycoproteins potently induced the NLRP3 inflammasome and GSDMD-dependent pyroptosis with subsequent IL-1β secretion in THP-1 macrophages. Our findings are of particular importance to understand viral pathogenesis and surface exposed glycoproteins, which seem to have a dual function as mediators of viral entry and inflammasome activation. Furthermore, our results can foster vaccine development, since most antigens used so far also impact innate immunity.

## 2. Materials and Methods

### 2.1. Cell Lines and Cell Culture

THP-1 cells, a human monocytic cell line, were cultured in RPMI medium (Thermo Fisher Scientific, Waltham, MA, USA) containing 10% FBS (PAN Biotech, Aidenbach, Germany). For differentiation into macrophages cells were incubated with 20 nMol Phorbol 12-myristate 13-acetate (PMA) (Merck, Darmstadt, Germany) for 24 h. After 24 h, medium was exchanged, and cells were cultured for another 24 h without PMA. All cells were cultured at 37 °C with 5% CO_2_.

### 2.2. Stimulation of THP-1 Macrophages

The medium was exchanged, and cells were incubated with DMSO (Merck; solvent of inhibitors) or MCC950 (Merck; 10 µM) for 2 h. LPS (Merck; 5 µg/mL) and viral glycoproteins (10 µg/mL) were added, and cells were incubated for another 4 h for inflammasome priming. Nigericin (Merck; 5 µM) or adenosine triphosphate (ATP) (Thermo Fisher Scientific; 5 mM) were added and incubated for 2 h for inflammasome activation. For ELISA, the supernatant was transferred to a new plate and stored at −80 °C. For cell death analysis cells were incubated another 24 h with nigericin.

### 2.3. CRISPR-Cas9 Knockout Cell Line Generation

THP-1 macrophages expressing Cas9 and eGFP (kindly provided by Prof. Michael Hallek, University Hospital of Cologne) were resuspended at a concentration of 10^6^ cells/mL in RPMI with 10% FBS and 1% penicillin streptomycin (Thermo Fisher Scientific). An amount of 20 mM HEPES buffer solution (Thermo Fisher Scientific) and lentiviral vectors with guide RNAs (LentiArray CRISPR Guide RNA; Thermo Fisher Scientific) for GSDMD and NLRP3 were added (MOI = 5). Tubes were centrifuged (800 rpm, 2 h, 37 °C) for spin infection. Transduced cells were seeded into 6-well plates and incubated for 24 h. After 24 h the medium was changed. Cells were expanded and 4 days after transduction puromycin (ROTH, Karlsruhe, Germany; 1 µg/mL) was added. Efficacy of knockout was confirmed by Western Blot analysis.

### 2.4. IL-1β ELISA

Human IL-1 beta Uncoated ELISA Kit (Thermo Fisher Scientific) was used following manufacturer’s instructions. All supernatants were diluted 1:50 in ELISA diluent. The samples were measured using a Hidex sense microplate reader (Hidex, Turku, Finland) at OD 450 nm–570 nm. Sample concentrations were determined using the provided standard curve (range: 2–150 pg/mL).

### 2.5. Immunoblot Analysis

#### 2.5.1. Preparation of Supernatants

Cells were stimulated as stated above. The supernatant was transferred and prepared using methanol (ChemSolute, Renningen, Germany) and chloroform (AppliChem, Darmstadt, Germany). Pellet was suspended in loading buffer (100 mM Tris-HCL/4% sodium dodecyl sulfate/0.2% bromophenol blue/20% glycerol/ 200 mM β-mercaptoethanol) and incubated at 95 °C for 5 min.

#### 2.5.2. Preparation of Cell Lysates

After stimulation, cell culture plates were stored on ice and RIPA buffer (Thermo Fisher Scientific) was used to lyse the cells. Samples were centrifuged (16,000× *g*, 4 °C, 20 min) and the supernatant was used for analysis. Pierce BCA Protein Assay Kit (Thermo Fisher Scientific) was performed to determine protein concentrations. Samples were diluted in loading buffer and incubated at 95 °C for 5 min.

#### 2.5.3. Immunoblot

Samples and markers were applied to gels, which were run at 120 V. Subsequently, gels were blotted using the Trans-Blot Turbo Transfer System (BioRad, Berkely, CA, USA). Membranes were blocked for 1 h and primary antibodies ASC (B-3) (Santa Cruz Biotechnology, INC., Dallas, TX, USA; 1:500 in 5% dried milk), NLRP3 (Cell Signaling Technology, Danvers, MA, USA; 1:1000 in 5% BSA in TBST buffer), P-NFkB (Cell Signaling Technology; 1:1000 in 5% dried milk), cleaved Interleukin-1β (Cell Signaling Technology; 1:1000 in 5% dried milk), IL-1β (Cell Signaling Technology; 1:1000 in 5% BSA in TBST buffer), cleaved GSDMD (Cell Signaling Technology; 1:500 in 5% dried milk) or GSDMD (Merck; 1:500 in 5% dried milk) were added and incubated at 4 °C overnight. After washing, anti-mouse IgG or anti-rabbit IgG (both Cell Signaling Technology; 1:5000 in 5% dried milk) were added for 1 h. Membranes were developed using the Super Signal West Femto Maximum Sensitivity Substrate (Thermo Fisher Scientific) and visualized using the FUSION SOLO S imaging system (Vilber Lourmat, Eberhardzell, Germany).

### 2.6. Flow Cytometry

Cells were stimulated and incubated as described before. FITC Annexin V Apoptosis Detection Kit with PI (BioLegend, San Diego, CA, USA) were used according to manufacturer’s instructions. Stained cells were analyzed using the MACS Quant flow cytometer (Miltenyi Biotec, Bergisch Gladbach, Germany).

### 2.7. Immunofluorescence Staining

Cells were plated into 8-well chamber culture slides at 6 × 10^4^ cells/well, differentiated with PMA and stimulated as described before. Cells were washed with PBS (Thermo Fisher Scientific), fixed with 4% PFA for 15 min, washed and incubated with 5% FBS/0.1% Tween/0.1% Triton/PBS for 1 h. For ASC speck staining, cells were incubated overnight with the ASC Antibody (B-3) Alexa Fluor 488 (Santa Cruz Biotechnology, INC.; 1:100) in 3% FBS/0.1% Tween/0.1% Triton/PBS at 4 °C. After washing with PBS, DAPI (Thermo Fisher Scientific; 1:1000) was added and cells were incubated for 10 min. Cells were washed with PBS and slides were mounted with ProLong Gold antifade reagent (Thermo Fisher Scientific). Slides were scanned using the Olympus FV1000 Microscope (Olympus, Shinjuku, Japan). Images were taken with the same microscope settings for all conditions. On average we acquired the following number of cells per condition per biological replicate: Ø: 126, LPS: 178, SP-CoV-2: 174, gB HCMV: 178, E2 HCV: 140. Three biological replicates were performed per condition.

### 2.8. LDH Assay

THP-1 macrophages were plated at 5 × 10^3^ cells/well, differentiated with PMA and stimulated. After 24 h CyQUANT LDH Cytotoxicity Assay (Thermo Fisher Scientific) was performed following the manufacturer’s instructions. Plates were measured with Hidex sense microplate reader at 490 nm–680 nm.

### 2.9. Protein Production

The plasmid for the SARS-CoV-2 spike protein (MN908947; AA: 1-1208; RRAR to GSAS; F817P; A892P; A899P; A942P; K986P; V987P) was kindly provided by Jason S. McLellan [23] and the SARS-CoV-1 plasmid (AAP13567; AA: 18-1190) was purchased from Sino Biological (Codon Optimized). The two coronavirus ectodomains (SARS-CoV-1 AA: 18-1190; SARS-CoV-2 AA: 1-1208) as well as the S2 region from SARS-CoV-2 (AA: 686-1208) were amplified from the synthetic gene plasmids by PCR and subsequently cloned into a modified sleeping beauty transposon expression vector containing a C-terminal Twin-Strep-Tag and verified by sequencing. In the case of the two ectodomains, a T4 foldon was added at the C-terminus. For the recombinant protein productions, stable HEK293 EBNA cell lines were generated employing the sleeping beauty transposon system [24]. Briefly, expression constructs were transfected into the HEK293 EBNA cells and after selection with puromycin, cells were expanded into triple flask and induced with doxycycline. Cell supernatants were filtered, passed through Strep-Tactin XT (IBA Lifesciences, Göttingen, Germany) resin, washed with 1 M NaCL/TBS, and eluted with biotin-containing TBS-buffer (IBA Lifesciences). After dialysis against TBS-buffer, the recombinant proteins were validated by SDS-PAGE followed by Coomassie staining. All reusable materials were treated with 1 M NaOH for 2 h and rinsed with ddH_2_O water to remove putative LPS contamination.

For gB HCMV and E2 HCV, codon-optimized synthetic cDNA corresponding to soluble C-terminally truncated versions of HCMV gB comprising residues [18] or HCV E2 isolate UKN2b_2.8 comprising residues 384–715 were cloned into a modified Drosophila S2 expression vector described previously and transfection was performed as reported earlier [25]. For large-scale production, cells were induced with 4 μM CdCl2 at a density of approximately 7 × 10^6^ cells/mL for 8 days, pelleted, and the soluble ectodomain was purified by affinity chromatography from the supernatant using a StrepTactin Superflow column followed by size exclusion chromatography using a Superdex200 column in 10 mM TRIS pH 8.0, 150 mM NaCl.

## 3. Results

### 3.1. Viral Glycoproteins Activate the NLRP3 Inflammasome

Viral host cell innate immune response varies broadly and it is known that several cellular inflammation-associated pathways can be activated by different viruses [9,10,26]. Therefore, we aimed to decipher the effect of purified viral glycoproteins of various viruses (SARS-CoV-2, SARS-CoV-1, HCMV, and HCV) on the activation of the NLRP3 inflammasome. We used glycoproteins of four different viruses to cover different forms of infections, such as acute vs. chronic.

**Figure 1:**
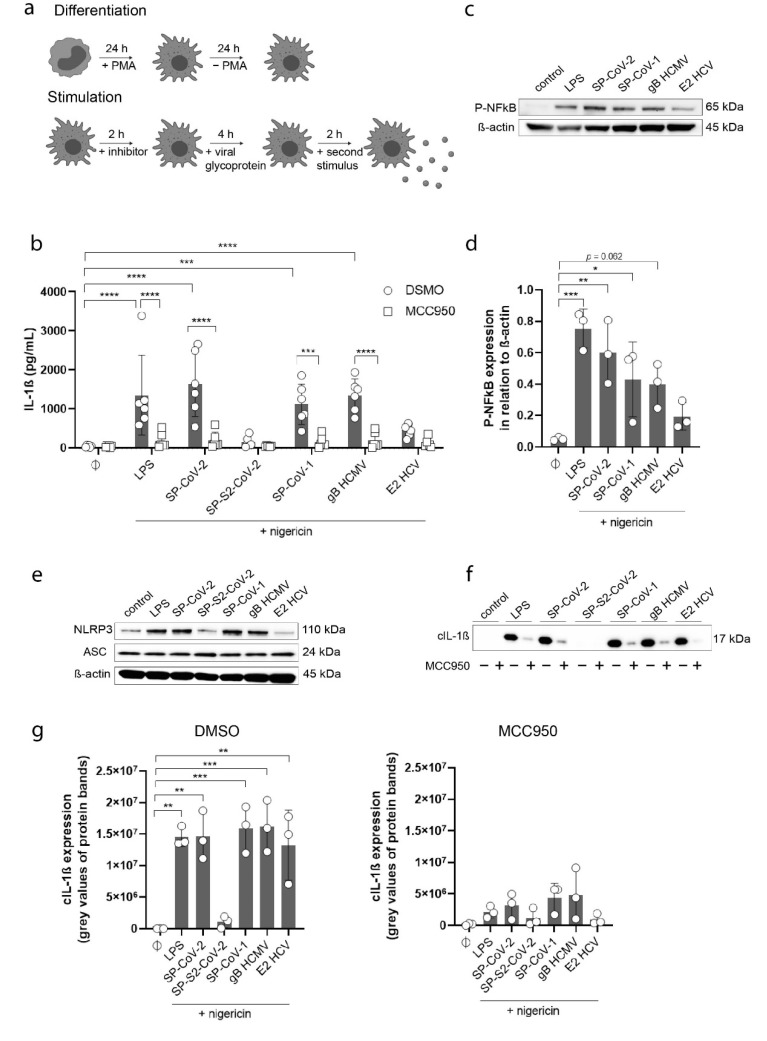
(**a**) Scheme of the experimental setup. THP-1 monocytes were differentiated using PMA (20 nMol) for 24 h and additionally incubated for 24 h without PMA. THP-1 macrophages were exposed to inhibitors for 2 h, subsequently stimulated with viral glycoproteins as priming signal for 4 h and as activation signal with nigericin for an additional 2 h. (**b**) Quantification of IL-1β concentration (pg/mL) in the supernatant of THP-1 macrophages stimulated with LPS (5 µg/mL) or viral antigens (as indicated, 10 µg/mL) in the presence (square) or absence (circle) of MCC950 (10 µM) (*n* = 6). All cells were stimulated with nigericin for 2 h (5 µM). Results are expressed as mean ± SD. Statistical analysis was done using two-way ANOVA with Tukey’s and Sidak’s multiple comparisons test (*** = *p* ≤ 0.001; **** = *p* ≤ 0.0001). (**c**) Detection of P-NFkB (1:1000 for P-NFkB antibody) in total cell lysates of THP-1 macrophages stimulated with LPS (5 µg/mL) or viral antigens (as indicated, 10 µg/mL). All cells were stimulated with nigericin (5 µM). ß-actin (1:1000 dilution) was used as a loading control. (**d**) Quantified results of Western Blot in Figure 1c are expressed as mean ± SD. Statistical analysis was performed using one-way ANOVA with Dunnett’s multiple comparisons test. Results were compared to a control (Ø) (* = *p* ≤ 0.05; ** *p* ≤ 0.01; *** *p* ≤ 0.001). (**e**) Detection of NLRP3 and ASC (1:1000 dilution for NLRP3 antibody, 1:500 dilution for ASC antibody; n = 3) in total cell lysates of THP-1 macrophages stimulated with LPS (5 µg/mL) or viral antigens (as indicated, 10 µg/mL). All cells were stimulated with nigericin (5 µM). ß-actin (1:1000 dilution) was used as a loading control. (**f**) Detection of cleaved IL-1ß (cIL-1ß) (n = 3) in the supernatant of THP-1 macrophages stimulated with LPS (5 µg/mL) or viral antigens (as indicated, 10 µg/mL) in the presence (+) or absence (-) of MCC950 (10 µM) (top). All cells were stimulated with nigericin (5 µM). (**g**) Quantified results of Western Blot in Figure 1d are expressed as mean ± SD. Statistical analysis was done using one-way ANOVA with Tukey’s multiple comparisons test (** *p* ≤ 0.01; *** *p* ≤ 0.001).

As an in vitro model, we used human THP-1 monocytes, which were differentiated to macrophages using PMA. We utilized a chemical genetics approach to analyze NLRP3 activation. MCC950, a well-known NLRP3 inhibitor [27], was used to pre-incubate THP-1 macrophages. Subsequently, THP-1 macrophages were stimulated with each viral glycoprotein as the first stimulus for inflammasome priming. In a second step, we incubated the cells with nigericin as an activation signal (Figure 1a).

As viral glycoproteins, SP-CoV-2, the spike protein of SARS-CoV-1 (SP-CoV-1), gB HCMV and the viral structure protein E2 HCV were used. The S2 subunit of SP-CoV-2 (SP-S2-CoV-2) which fails to bind plasma membranes was used as a negative control. SP-S2-CoV-2 is the membrane-fusion domain and does not prime the NLRP3 inflammasome in macrophages [22]. LPS, a well-known inflammasome activator, was used as positive control. To determine NLRP3 inflammasome activation, we measured IL-1β in the supernatants of THP-1 macrophages upon stimulation. LPS, SP-CoV-2, SP-CoV-1 and gB HCMV were able to significantly increase the concentration of IL-1β in the cell supernatant (Figure 1b). Increased IL-1β levels (11.2-fold increase) were also detected upon stimulation with E2 HCV. Chemical inhibition with MCC950 abrogated IL-1β secretion upon stimulation with viral glycoproteins (Figure 1b). As verification and to exclude unspecific effects by high protein concentrations, we stimulated THP-1 macrophages with a 10-fold lower protein concentration (1 µg/mL) and were able to see similar results, which occurred in a dose-dependent manner (Supplementary Figure S1a). NLRP3 inflammasome activation could also be induced by using ATP instead of nigericin after as a second signal for IL-1ß release (Supplementary Figure S1b). After the stimulation with viral glycoproteins without a second signal such as nigericin or ATP, no increase in IL-1ß levels could be detected (Supplementary Figure S1c).

To investigate the upregulation of an immune response, we first quantified phospho-NFkB (P-NFkB) in total cell lysates. We were able to detect an increase in P-NFkB after the exposure to LPS, SP-CoV-2, SP-CoV-1, and gB HCMV (Figure 1c,d). Then, we analyzed the molecular mechanism of NLRP3 activation by quantifying NLRP3 and ASC protein levels in total cell lysates of stimulated THP-1 macrophages. Here, we could detect increased NLRP3 levels after stimulation with LPS, SP-CoV-2, SP-CoV-1, and gB HCMV (Figure 1e and Supplementary Figure S1d) indicating increased transcription of NLRP3 upon viral glycoprotein stimulation. In contrast, ASC protein levels were not affected by the stimulation (Figure 1e and Supplementary Figure S1e). We then analyzed IL-1ß levels in total cell lysates and could detect an increase in proteins levels after stimulation with LPS, SP-CoV-2, SP-CoV-1, and gB HCMV (Supplementary Figure S1f,g). As a hallmark of inflammasome activation, we analyzed the presence of cleaved IL-1β (cIL-1β) in the supernatant of stimulated THP-1 macrophages using Western blot analysis (Figure 1f). Strikingly, a significant increase in cIL-1β could be detected after stimulation with LPS and all viral glycoproteins (Figure 1f,g), whereas MCC950 was able to effectively block the secretion of cIL-1β (Figure 1f,g). SP-S2-CoV-2, however, did not increase cIL-1β (Figure 1f,g).

**Figure 2:**
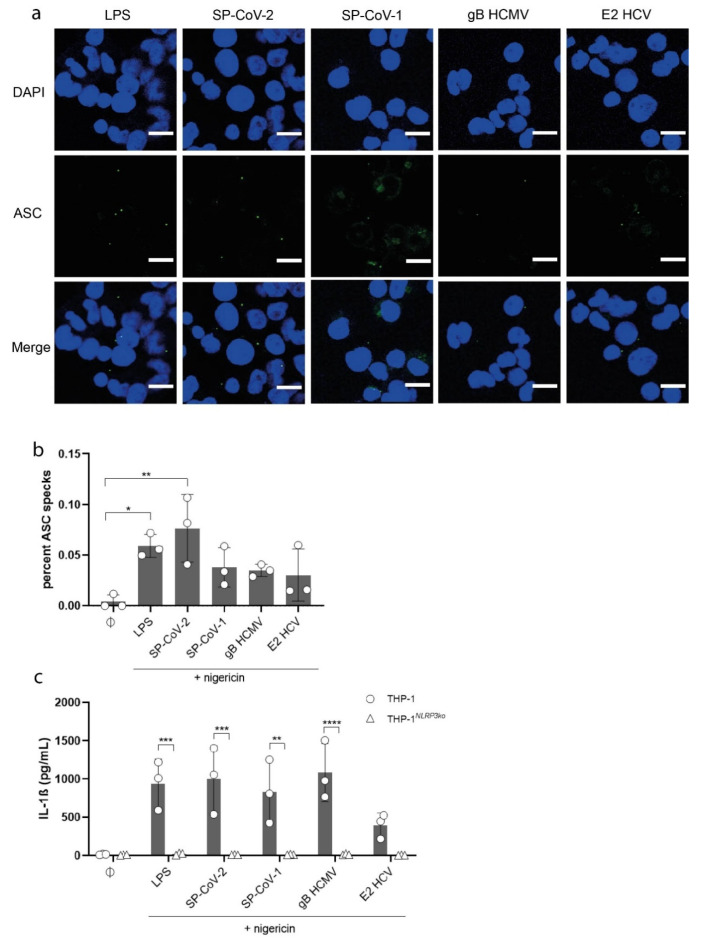
(**a**) Confocal fluorescence microscopy images of THP-1 macrophages stimulated with LPS (5 µg/mL) or viral antigens (as indicated, 10 µg/mL). All cells were stimulated with nigericin (5 µM). Cells were stained with ASC Antibody (B-3) Alexa Fluor 488 (1:100 dilution) and DAPI and analyzed with the same microscope settings (*n* = 3). Representative images are shown (scale bar = 15 µm). (**b**) Quantified results of fluorescence microscopy shown in Figure 2a are expressed as mean ± SD. Statistical analysis was performed using one-way ANOVA with Tukey’s multiple comparisons test (* = *p* ≤ 0.05; ** = *p* ≤ 0.01). (**c**) Quantification of IL-1β concentration (pg/mL) in the supernatant of THP-1 macrophages (circle) and THP-1*^NLRP3ko^* cells (triangle) stimulated with LPS (5 µg/mL) or viral antigens (as indicated, 10 µg/mL) (*n* = 3). All cells were stimulated with nigericin (5 µM). Results are expressed as mean ± SD. Statistical analysis was done using two-way ANOVA with Sidak’s multiple comparisons test (** = *p* ≤ 0.01; *** = *p* ≤ 0.001; **** = *p* ≤ 0.0001).

Next, we visualized ASC-SPECK formation, which is an important step in NLRP3-inflammasome activation via immunofluorescence microscopy of THP-1 macrophages stimulated with viral glycoproteins. ASC-SPECK formation indicates complete assembly of the inflammasome. The amount of ASC-SPECKS per cell (DAPI^+^) increased upon stimulation with LPS and the tested glycoproteins (Figure 2a,b).

Almost no specks were detected in unstimulated cells (Supplementary Figure S2a and Figure 2b). To confirm NLRP3 activation as the major driver of IL-1β secretion, we generated a THP-1 NLRP3 knockout cell line (THP-1^NLRP3ko^) by CRISPR-Cas9 technology (Supplementary Figure S2b). A stable Cas9 expressing THP-1 line was infected with lentiviral vectors encoding gRNAs against NLRP3. Wild-type THP-1 macrophages and THP-1^NLRP3ko^ cells were stimulated as previously described. While an increase in IL-1β secretion could be detected in wild-type cells, IL-1β levels in the supernatants of THP-1^NLRP3ko^ cells stimulated with LPS, SP-CoV-2, SP-CoV-1, gB HCMV or E2 HCV remained at baseline (Figure 2c). These results clearly confirm that viral glycoprotein stimulation of macrophages induces NLRP3 inflammasome activation and IL-1ß secretion.

### 3.2. Viral Glycoprotein Exposure Induces Necrotic Cell Death

**Figure 3:**
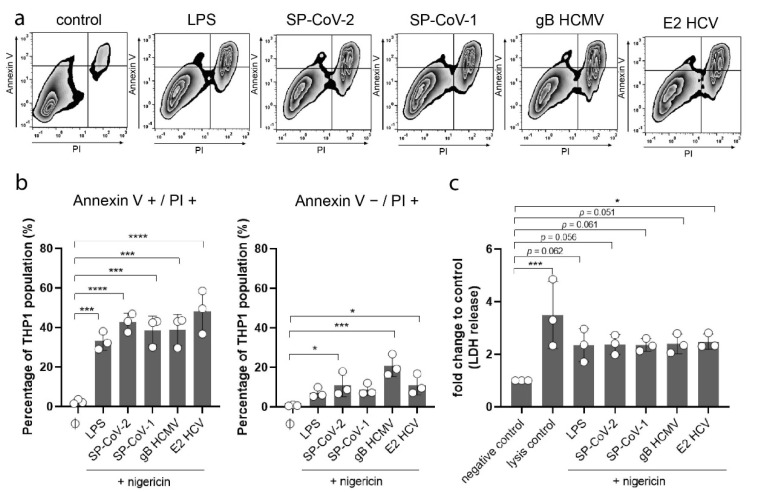
(**a**) THP-1 macrophages were stimulated with LPS (5 µg/mL) or viral antigens (as indicated, 10 µg/mL). All cells were stimulated with nigericin (5 µM). After 24 h cells were stained with Annexin V and PI. Cells were analyzed using flow cytometry (n = 3). Representative images are shown. (**b**) Quantified results of flow cytometry analysis shown in Figure 3a are expressed as mean ± SD. Statistical analysis was performed using one-way ANOVA with Dunnett’s multiple comparisons test. Results were compared to a control (Ø) (* = *p* ≤ 0.05; *** = *p* ≤ 0.001; **** = *p* ≤ 0.0001). (**c**) Quantification of lactate dehydrogenase (LDH) in the supernatant of THP-1 macrophages stimulated with LPS (5 µg/mL) or viral antigens (as indicated, 10 µg/mL) was performed 24 h after stimulation (n = 3). All cells were stimulated with nigericin (5 µM). Results are expressed as mean ± SD. Statistical analysis was performed using one-way ANOVA with Dunnett’s multiple comparisons test. Results were compared to a control (negative control). (* *p* ≤ 0.05; *** *p* ≤ 0.001).

Necrotic cell death, in particular pyroptosis, is closely linked to inflammasome activation. To draw that connection, we performed an Annexin V and PI cell death staining on macrophages to analyze if viral glycoproteins can induce cell death. THP-1 macrophages were stimulated as stated above and incubated for an additional 24 h. Cells were then stained with Annexin V and PI and analyzed using flow cytometry (representative images and gating strategy can be found in Figure 3a and Supplementary Figure S2c).

We could detect a significant increase in cells undergoing late apoptosis/necrosis (Annexin V+/PI+) after the stimulation with LPS or with the respective viral glycoproteins (Figure 3b). Interestingly, we could also detect a significant increase in necrosis (Annexin V-/PI+) in cells exposed to SP-CoV-2, gB HCMV, and E2 HCV (Figure 3b). To further investigate necrotic cell death, we performed an LDH releasing assay on stimulated THP-1 macrophages after 24 h of incubation. Compared to the negative control we detected an increase in LDH levels in the supernatant of cells exposed to LPS, SP-CoV-2, SP-CoV-1, gB HCMV, and E2 HCV (Figure 3c). These results indicate that viral glycoproteins could induce necrotic cell death.

### 3.3. Viral Glycoproteins Induce GSDMD Dependent Pyroptosis

**Figure 4:**
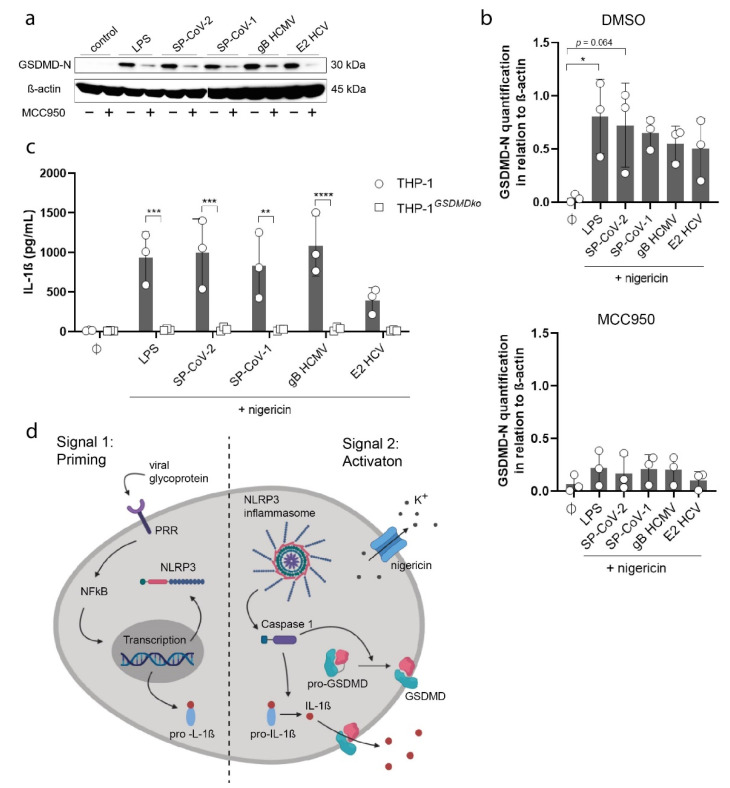
(**a**) Detection of cleaved GSDMD (GSDMD-N) in cell lysates of THP-1 macrophages stimulated with LPS (5 µg/mL) or viral antigens (as indicated, 10 µg/mL) in the presence (+) or absence (-) of MCC950 (10 µM). All cells were stimulated with nigericin (5 µM). (**b**) Quantified results of Western Blot shown in Figure 4a are expressed as mean ± SD. Statistical analysis was performed using one-way ANOVA with Tukey’s multiple comparisons test (* *p* ≤ 0.05). (**c**) Quantification of IL-1β concentration (pg/mL) in the supernatant of THP-1 macrophages (circle) and THP-1*^GSDMDko^* cells (square) stimulated with LPS (5 µg/mL) or viral antigens (as indicated, 10 µg/mL) (n = 3). All cells were stimulated with nigericin (5 µM). Bars indicate mean ± SD. Statistical analysis was performed using two-way ANOVA with Sidak’s multiple comparisons test (** = *p* ≤ 0.01; ***, *p* ≤ 0.001; ****, *p* ≤ 0.0001). (**d**) Scheme of NLRP3 activation and induction of pyroptosis by viral antigens.

NLRP3 inflammasome activation and GSDMD dependent pyroptosis are closely interconnected and known as a cause of cell death of macrophages upon exposure to viral or bacterial pathogens [28,29]. Cleavage of GSDMD by caspase-1 represents an important step in pyroptotic cell death, thus we performed immunoblots of cleaved GSDMD (GSDMD-N) in total cell lysates of stimulated THP-1 macrophages (Figure 4a).

Here, we could detect a strong increase of GSDMD-N levels in cell lysates of cells stimulated with LPS and with all viral glycoproteins (Figure 4b). As expected, MCC950 diminished GSDMD-N levels efficiently (Figure 4b). We also detected GSDMD in total cell lysates and could not detect an increase in protein expression after stimulation (Supplementary Figure S2d,e). To confirm the induction of pyroptosis via GSDMD cleavage, we generated THP-1 GSDMD knockout cells (THP-1^GSDMDko^) (Supplementary Figure S2b). In comparison to wild-type THP-1 macrophages, THP-1^GSDMDko^ stimulated with LPS or viral glycoproteins failed to secrete IL-1β (Figure 4c).

In summary, we show that several viral glycoproteins act as viral PAMPs that potently induce NLRP3 inflammasome activation and consequently pyroptotic cells death in THP-1 macrophages (Figure 4d).

## 4. Discussion

Inflammasomes, as an essential part of the innate immune response, are of particular importance for the host defense against various pathogens. While the role of inflammasomes in the antiviral response is well described, in-depth knowledge on exact triggers and structural components activating associated signaling pathways is scarce [9,10,30,31]. In this paper, we were able to show that surface exposed glycoproteins of several viruses can function as activators of the NLRP3 inflammasome and pyroptotic cell death.

Several viruses have been connected with inflammasome activation before. Huang et al. show that the AIM2 inflammasome is activated in THP-1 macrophages when exposed to CMV [32]. When exposed to HCV genotype 2a, THP-1 macrophages, primary macrophages and Kupffer cells show an increase in the secretion of the inflammatory cytokine IL-1β [33]. Most recently, the inflammasome has been discussed as part of the inflammation process in COVID-19 and is associated with severe outcomes in patients [13]. While these studies confirm an interaction between whole viruses and inflammasomes, especially the AIM2 inflammasome, the influence of surface-associated glycoproteins as triggers of the innate immune system is not well studied.

Detection of invading viruses involves PRRs. These receptors can bind to several viral components such as viral RNA, viral DNA, virus coat proteins, or viral glycosylated proteins [9]. In this paper, we show that glycoproteins of several important viruses are capable of priming the NLRP3 inflammasome. All proteins were able to induce the secretion of cleaved IL-1β, which represents the hallmark for inflammasome activation. For further evidence, we showed that the glycoproteins are able to trigger the formation of ASC-SPECKS as the proof of NLRP3 inflammasome formation. Furthermore, we could also demonstrate increased NLRP3 protein levels after stimulation with the viral glycoproteins. Why E2 HCV was able to induce secretion of cleaved IL-1β without strong upregulation of NLRP3 requires further analysis. In fact, our data strongly links virus-related inflammasome activation, as discussed above, to the corresponding viral glycoproteins, which activate host inflammasome pathways and therefore act as viral PAMP for the innate immune system. Further research is required to understand which receptors and signaling events leads to NFkB and NLRP3 activation. While we have focused on the most studied and well-described inflammasome complex (NLRP3), we cannot rule out that stimulation with viral glycoproteins can also activate other inflammasome complexes.

Studies surrounding viral glycoproteins and their effects on innate immunity are even more important, as they are increasingly exploited as key components of vaccine constructs. A prominent example is the COVID-19 vaccine. So far, all approved vaccines use the SARS-CoV-2 spike protein as a target antigen [17]. For HCMV infections, the most promising vaccine candidate in development is a soluble gB subunit vaccine [34]. For HCV infections several vaccine candidates are currently examined and potential targets are nonstructural proteins, HCV E1, E2, and core proteins [35]. Several studies have investigated the immune response to vaccines. As a link between innate and adaptive immunity, they show an interaction between vaccine components and PRRs leading to chemokine secretion and subsequent attraction of innate immune cells [36,37]. This highlights the importance of our findings, which show that all studied proteins are potent triggers of innate immune signaling via the inflammasome. It is highly conceivable that the observed NLRP3 inflammasome activation and subsequent IL-1β secretion is important for a vaccine-induced immunity, which requires further confirmation in additional in vivo and ex vivo studies, or clinical trials. Our analyses are based on in vitro experiments with a monocytic cell line which allows for genetic manipulation. At least for SARS-CoV-2 we were able to show that primary human macrophages derived from COVID-19 patients also activate the NLRP3 inflammasome upon spike protein exposure [22]. We will now analyze NLRP3 activation in primary macrophages derived from seropositive or seronegative individuals for the other viruses.

Currently, we cannot rule out that the glycosylation pattern of the viral glycoproteins could have an impact on inflammasome formation and activation. However, crystal structures for all glycoproteins have been published and all affinity purified proteins have been shown to induce immune responses in vitro and in vivo [18,38]. Usage of different producer cells such as HEK293 cells and drosophila S2 insect cells as performed in our study rules out expression system dependent artefacts with regard to glycosylation pattern.

Furthermore, we analyzed the inflammatory cell death pyroptosis, as one key component in the defense against viral pathogens. Pyroptosis was first described in bacterial infections, e.g., with *Shigella*, *Salmonella,* and *Yersinia* [39]. However, in more recent studies pyroptosis has also been linked to viral pathogens [29,30]. An example of pyroptosis in viral infections is the inflammatory necrosis in cultured liver cells following the infection with HCV [40]. Here we provide evidence that pyroptosis can be induced by viral glycoproteins in THP-1 macrophages. All proteins investigated in this study induce necrotic cell death in THP-1 macrophages, which could be abrogated by a GSDMD knockout.

It is not clear whether the described inflammasome activation and the induction of pyroptosis will be beneficial to the host, e.g., by promoting viral clearance, or whether detrimental consequences such as tissue damage predominate. These questions are crucial when analyzing potential therapeutics interfering with innate immunity. As an example, in COVID-19, IL-1β has been linked to hyperinflammation with respiratory failure and blocking IL-1 has been studied as a therapeutic approach [41,42]. In HCV infections, IL-1 is discussed to have antiviral activity and an important role in viral clearance in acute infections [43]. However, IL-1ß is also discussed as a potential stimulus for inflammation and possibly consequent liver fibrosis in chronic HCV infections [33]. This demonstrates that there are still a lot of uncertainties associated with IL-1β and its role in viral infections and innate immune responses. Confirmation of our findings in in vivo models of infection or vaccination will help to answer these open questions. We provide new impulses for further investigation of viral glycoproteins as innate immunity triggers as a starting point for these studies.

## Data Availability

The data presented in this study are available on request from the corresponding author. All generated data is represented within this manuscript.

## Supplementary Materials

The following are available online at https://www.mdpi.com/article/10.3390/v13102076/s1, Supplementary Figure S1. (a) Quantification of IL-1ß concentration (pg/mL) in the supernatant of THP-1 macrophages stimulated with LPS (5 µg/mL; n = 6) or viral antigens (as indicated, 1 µg/mL; n = 3). All cells were stimulated with nigericin (5 µM). Statistical analysis was performed using one-way ANOVA with Tukey’s multiple comparisons test. (* = *p* ≤ 0.05). (b) THP-1 macrophages were stimulated with LPS (5 µg/mL) or viral glycoproteins (as indicated, 10 µg/mL) and subsequently exposed to ATP (5 mM) (n = 3). IL-1ß concentration (pg/mL) was quantified in supernatant via IL-1ß ELISA. (c) THP-1 macrophages were stimulated with viral glycoproteins (as indicated, 10 µg/mL). IL-1ß concentration (pg/mL) was quantified in supernatant via IL-1ß ELISA. (d) Quantification of NLRP3 Western Blot shown in Figure 1e. NLRP3 expression was quantified in relation to ß-actin expression. (e) Quantification of ASC Western Blot shown in Figure 1e. ASC expression was quantified in relation to ß-actin expression. (f) Detection of IL-1ß (1:1000 for IL-1ß antibody) in total cell lysates of THP-1 macrophages stimulated with LPS (5 µg/mL) or viral antigens (as indicated, 10 µg/mL). All cells were stimulated with nigericin (5 µM). ß-actin (1:1000 dilution) was used as a loading control. (g) Quantified results of Western Blot in Figure S1f are expressed as mean ± SD. Statistical analysis was performed using one-way ANOVA with Dunnett’s multiple comparisons test. Results were compared to a control (Ø) (**, *p* ≤ 0.01). Supplementary Figure S2. (a) Fluorescence microscopy of unstimulated THP-1 macrophages (control). Cells were stained with ASC Antibody (B-3) Alexa Fluor 488 (1:100 dilution) and with DAPI. Representative images are shown (scale bar: 15 µm). (b) Scheme of the generation of knockout (KO) cell lines. THP-1 macrophages were first transduced with Cas9-GFP. Cells were then transduced with guide RNAs (gRNA). (c) Representative gating strategy showing flow cytometry analysis for THP-1 cell death staining with Annexin V and PI. (d) Detection of GSDMD (1:500 for GSDMD antibody) in total cell lysates of THP-1 macrophages stimulated with LPS (5 µg/mL) or viral antigens (as indicated, 10 µg/mL). All cells were stimulated with nigericin (5 µM). ß-actin (1:1000 dilution) was used as a loading control. (e) Quantified results of Western Blot in Figure S2d are expressed as mean ± SD.
