# Supplementary material for: Viral Glycoproteins Induce NLRP3 Inflammasome Activation and Pyroptosis in Macrophages
Source: Viruses. 2021 Oct 15;13(10):2076. doi: 10.3390/v13102076 (PMC8538122; doi:10.3390/v13102076)
Supplement: Supplementary file 1 [file viruses-13-02076-s001.zip › viruses-1398154-supplementary material.pdf]

Supplementary Materials:

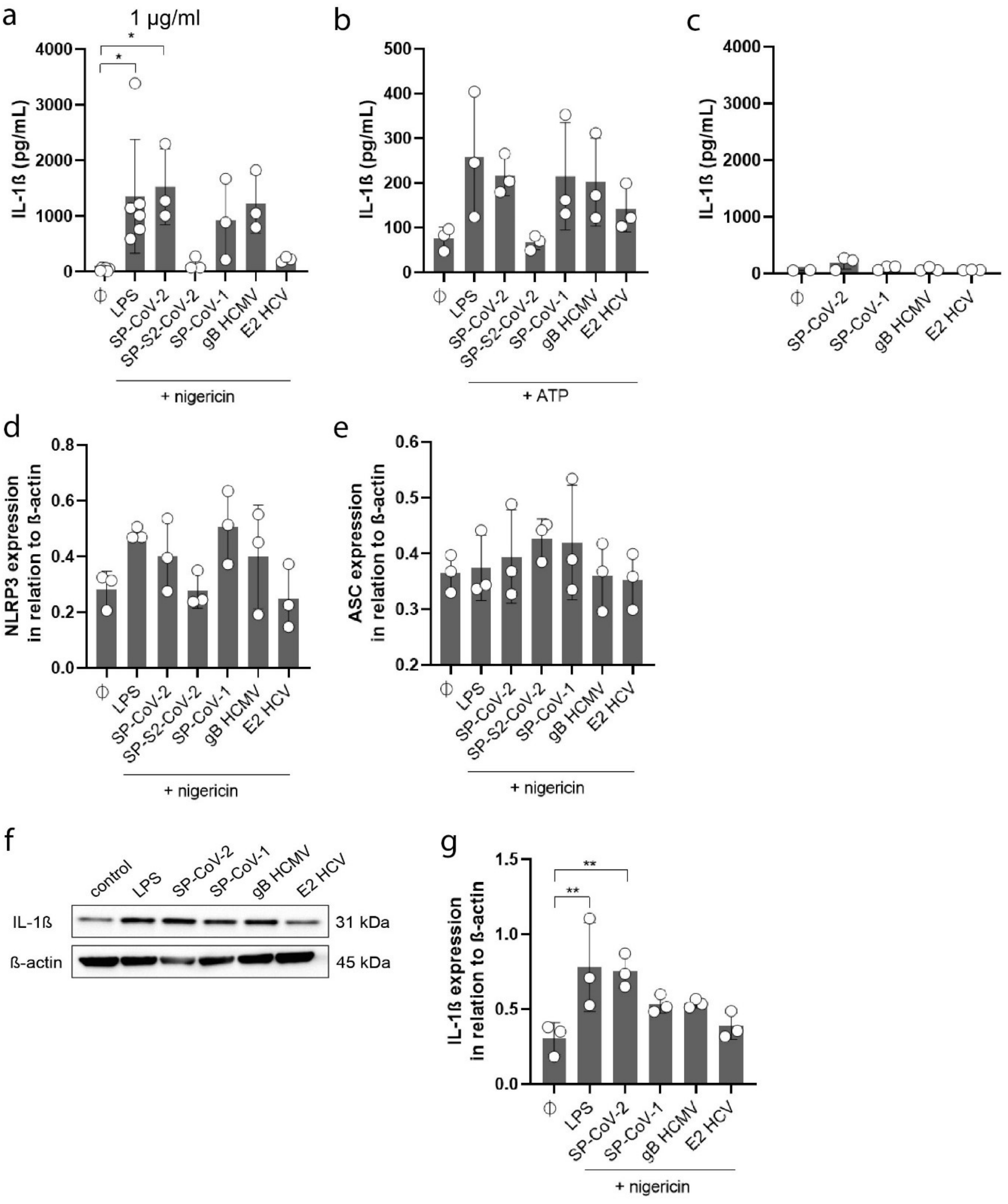

Figure S1. **(a)** Quantification of IL-1ß concentration (pg/ml) in the supernatant of THP-1 macrophages stimulated with LPS (5 µg/ml; n= 6) or viral antigens (as indicated, 1 µg/ml; n= 3). All cells were stimulated with nigericin (5 µM). Statistical analysis was done using one-way ANOVA with Tukey's multiple comparisons test. (* = $p \leq 0.05$). **(b)** THP-1 macrophages were stimulated with LPS (5 µg/ml) or viral glycoproteins (as indicated, 10 µg/ml) and subsequently exposed to ATP (5 mM) (n= 3). IL-1ß concentration (pg/ml) was quantified in supernatant via IL-1ß ELISA. **(c)** THP-1 macrophages were stimulated with viral glycoproteins (as indicated, 10 µg/ml). IL-1ß concentration (pg/ml) was quantified in supernatant via IL-1ß ELISA. **(d)** Quantification of NLRP3 western blot shown in Figure 1e. NLRP3 expression was quantified in relation to ß-actin expression. **(e)** Quantification of ASC western blot shown in Figure 1e. ASC expression was quantified in relation to ß-actin expression. **(f)** Detection of IL-1ß (1:1000 for IL-1ß antibody) in total cell lysates of THP-1 macrophages stimulated with LPS (5 µg/ml)

or viral antigens (as indicated, 10 µg/ml). All cells were stimulated with nigericin (5 µM). ß-actin (1:1000 dilution) was used as a loading control. **(g)** Quantified results of Western Blot in Supplementary Fig. 1f are expressed as mean ± SD. Statistical analysis was done using one-way ANOVA with Dunnett's multiple comparisons test. Results were compared to a control (Ø) (**, p≤ 0.01).

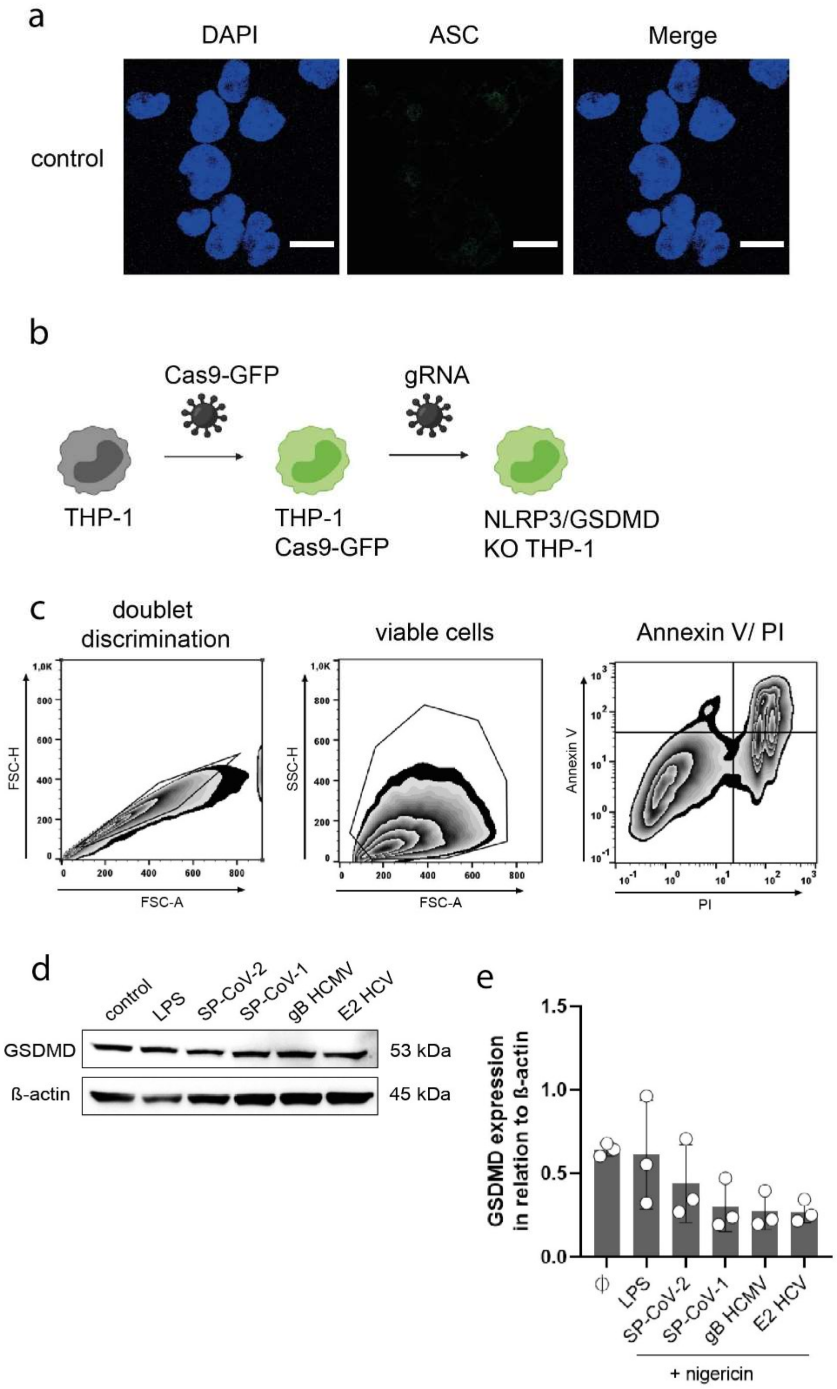

Figure S2. **(a)** Fluorescence microscopy of unstimulated THP-1 macrophages (control). Cells were stained with ASC Antibody (B-3) Alexa Fluor 488 (1:100 dilution) and with DAPI. Representative images are shown (Scale bar: 15 µm). **(b)** Scheme of the generation of knockout (KO) cell lines. THP-1 macrophages were first transduced with Cas9-GFP. Cells were then transduced with guide RNAs (gRNA). **(c)** Representative gating strategy showing flow cytometry analysis for THP-1 cell death staining with Annexin V and PI. **(d)** Detection of GSDMD (1:500 for GSDMD antibody) in total cell lysates of THP-1 macrophages stimulated with LPS (5 µg/ml) or viral antigens (as indicated, 10 µg/ml). All cells were stimulated with nigericin (5 µM). ß-actin (1:1000 dilution) was used as a loading control. **(e)** Quantified results of Western Blot in Supplementary Fig. 2d are expressed as mean ± SD.
